# Protective Capacity of *Helichrysum italicum* Infusion Against Intestinal Barrier Disruption and Translocation of *Salmonella* Infantis

**DOI:** 10.3390/ph17101398

**Published:** 2024-10-19

**Authors:** Katja Kramberger, Katja Bezek Kranjc, Zala Jenko Pražnikar, Darja Barlič-Maganja, Saša Kenig

**Affiliations:** Faculty of Health Sciences, University of Primorska, Polje 42, 6310 Izola, Slovenia; katja.kramberger@fvz.upr.si (K.K.); katja.kranjc@fvz.upr.si (K.B.K.); zala.praznikar@upr.si (Z.J.P.); darja.maganja@fvz.upr.si (D.B.-M.)

**Keywords:** *Helichrysum italicum*, *Salmonella* Infantis, intestinal barrier, tight junctions, inflammation

## Abstract

Background: *Helichrysum italicum* is a Mediterranean plant with well-known anti-inflammatory activity, but our previous whole transcriptome analysis has found that *H. italicum* infusion (HII) can also affect cytoskeletal rearrangement and tight junctions. The goal of the present study was to determine if HII improves the intestinal barrier (IB) dysfunction and by what mechanism. Methods: Caco-2 cells on Transwell inserts were used as a model of IB permeability. Heat-killed (HKB) or live *Salmonella* Infantis bacteria were used to induce IB integrity disruption upon three different testing conditions: pre-, co-, and post-treatment with 0.2 v/v% HII. Transepithelial electrical resistance values were used as an indicator of monolayer integrity before and after all treatments, and RT-PCR was used to assess the expression of tight junction proteins (TJPs) and inflammatory cytokines known to regulate intestinal permeability. Results: We found that all three treatments with HII improved the HKB-induced integrity disruption and decreased the down-regulation of *TJP1*, *OCLN*, and *CLDN1*, with the greatest effect observed in the pre-treated cells. Treatment with HII also decreased the up-regulation of *CLDN2*, *TNF-α*, *IL-1β*, and *IL-6*. In addition, pre-treatment of Caco-2 cells with HII prevented translocation of *S.* Infantis but did not prevent adhesion and invasion. Conclusion: This study showed that HII can improve inflammation-disrupted IB function by indirect modulation of mRNA expression of TJPs, especially in a preventive manner.

## 1. Introduction

The intestinal epithelium is a single-cell layer that serves as the largest and most important barrier against the external environment. The intestinal mucosal mechanical barrier primarily consists of intestinal epithelial cells and their tight junctions (TJs), which modulate selective permeability and physiologic barrier function through transcellular and paracellular pathways, respectively [1]. In addition, components such as immune cells, the intestinal microbiota, and anti-microbial peptides have crucial roles in supporting appropriate intestinal barrier (IB) function. A disturbance in the function of the IB leads to increased intestinal permeability, which in turn facilitates the translocation of harmful substances into the bloodstream and triggers a systemic inflammatory response. A dysfunctional IB is associated with a variety of diseases such as bacterial intestinal infections, inflammatory bowel disease, irritable bowel syndrome, diabetes, obesity, and food allergies [2]. Although it is not yet clear, whether the loss of IB integrity is the cause or the consequence of the ongoing inflammation associated with these conditions, targeting the IB permeability holds promise for the treatment and even prevention of disease onset [3].

Commensal microorganisms play a protective role in the digestive mucosa by participating in maintaining the physiological integrity of TJs, which preserve their barrier function during entero-pathogen challenges [4]. Among them, probiotics have been reported to potentiate the tightening of TJs, which improve the impermeability of the gut mucosa, counteracting the deleterious action of pathogens upon the TJ complex [5]. In contrast, some pathogenic microorganisms have developed strategies to disorganize the TJs, aiming to translocate through the digestive mucosa and invade their host. These entero-pathogens include entero-invasive or entero-toxigenic bacteria such as *Shigella*, *Salmonella*, *Escherichia coli*, *Yersinia*, *Campylobacter*, or *Vibrio cholerae* [6]. Genetic susceptibility, environmental factors, dietary habits, and changes in the composition of the gut microbiota can affect the intestinal epithelial and vascular barrier, facilitating bacterial translocation and endotoxemia [7]. Bacterial translocation is defined as the passage of viable bacteria from the gastrointestinal tract to the lamina propria and, subsequently, to the mesenteric lymph nodes and other extra-intestinal organs, such as the spleen, liver, or systemic circulation [8]. However, bacterial translocation also includes commensal organisms that transfer and engraft from one body site to another, where they potentially cause adverse health effects in the new environment [9]. 

Endotoxins such as lipopolysaccharides (LPS), the major components of the outer membrane of Gram-negative bacteria, have been identified as potent inducers of inflammation that can initiate severe systemic effects [10]. Although LPS may originate from skin and mucous membranes or local sites of bacterial infection, for instance, the gut microbiota is considered the main natural reservoir of pro-inflammatory endotoxins in the body [11]. Endotoxins are released when bacteria die, and then dissociated endotoxins are able to cross the gastrointestinal barrier to end up in the bloodstream [12]. Although LPS is detectable at low concentrations in the circulation of healthy individuals, there is evidence that LPS levels transiently increase following ingestion of fat-rich meals. For instance, in mice fed with a four-week high-fat diet, plasma levels of LPS were similar to those observed following a four week subcutaneous infusion of 300 µg/kg/day of LPS [13]. Such endotoxemia is defined as “metabolic endotoxemia” and has been proposed as a major cause of inflammation, including chronic low-grade inflammation [14]. Persistent inflammatory stimulation induces functional impairment of the intestinal TJ barrier that further generates pro-inflammatory cytokines, thereby perpetuating chronic inflammation [15]. Therefore, approaches that can alleviate inflammatory response and/or restore IB integrity may present promising strategies for therapeutic interventions in gut-related diseases.

Dietary components, including probiotics, polyphenols, lipids, and proteins, can improve IB function by either suppressing inflammation or by acting directly on the IB [16]. *Helichrysum italicum* is an aromatic Mediterranean herb rich in polyphenolic compounds and other secondary metabolites [17]. The genus *Helichrysum* Mill. (Asteraceae) is very complex, as it comprises cca. 500–600 species, which are geographically distributed also beyond the Mediterranean basin and thus diverse with respect to both phenotype and metabolite profile. Although the therapeutic potential of *H. italicum* in inflammatory disorders and infective diseases has been known since ancient times [18], the bioactivity of different extracts is still being intensively investigated today [19]. Infusions and decoctions of *H. italicum* are the most commonly used preparations in traditional medicine; however, limited scientific data on their bioactivity are available. The health benefits of the polyphenols, the main chemical class of compounds present in the *Helichrysum* species, are mainly attributed to their antioxidant and anti-inflammatory properties but were recently recognized also for their prebiotic activity [20]. The former two activities can be limited *in vivo* due to their poor bioavailability, but evidently, systemic action is also possible. In addition to established *in vitro* antioxidant activity [21] of *H. italicum* infusion (HII), in our previous studies, we have also demonstrated antioxidant [22] and anti-inflammatory effects [23] of HII in patients with traits of metabolic syndrome. Four week daily consumption of HII improved serum antioxidant properties and blood lipid profile and significantly reduced serum levels of pro-inflammatory markers (IL-6, IL-1β, and MCP-1), whereas levels of an anti-inflammatory IL-33 were increased, in addition to the beneficial effect on anthropometric traits [22,23]. Furthermore, serum levels of zonulin, an indirect marker of gut barrier permeability, were reduced, alongside the observed trend of bacterial phyla Proteobacteria and Firmicutes reduction, indicating improvement of gut dysbiosis [23]. A microarray-based transcriptome analysis of primary colon fibroblasts was performed to explore the mechanism of HII’s action. We discovered altered expression of genes involved in cytoskeletal rearrangement and cellular growth, enrichment of pathways such as interleukin signaling, and transcriptional regulation by TP53 [24]. Overall results suggested that HII could affect inflammation and metabolic diseases indirectly by maintaining the integrity of the IB, as well as directly via the regulation of genes involved in these processes.

In the present study, we aimed to further explore the action of HII on the IB using a Caco-2 cell monolayer and heat-killed *Salmonella* Infantis bacteria (HKB) as an inductor of cell inflammatory response. We evaluated the effects of HII on transepithelial electrical resistance (TEER), tight junction protein (TJP), inflammatory cytokine-coding gene expression, and the adhesion, invasion, and translocation capacity of *S.* Infantis. The results of our study suggest an important role of HII in maintaining the integrity of the IB, especially in inflammatory conditions. 

## 2. Results

### 2.1. Cytotoxicity of HII and HKB

**Figure 1:**
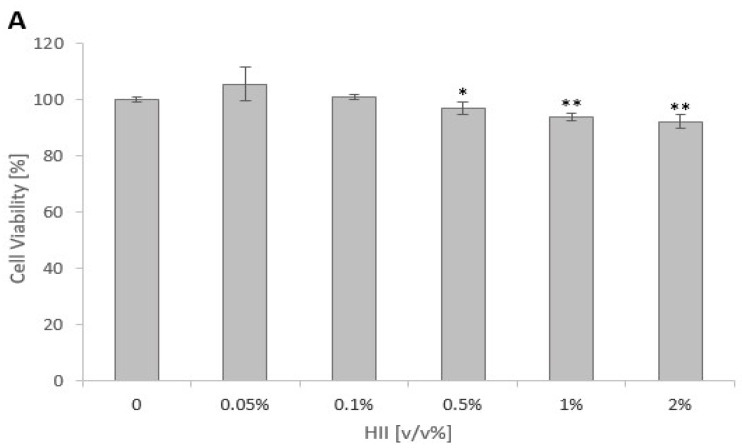

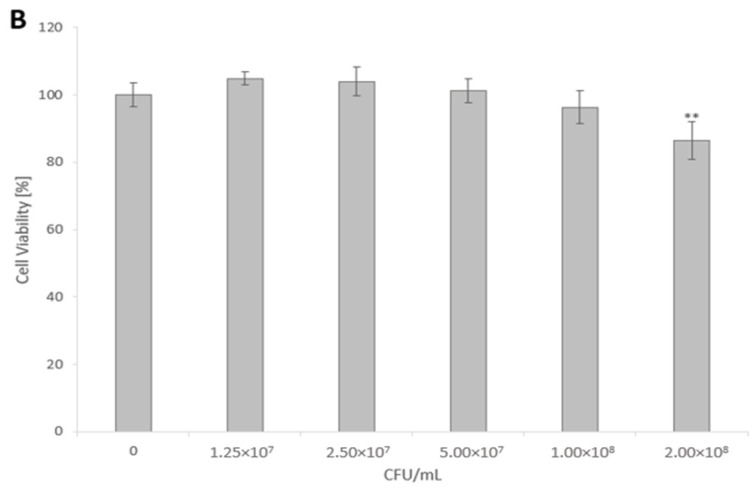
Cytotoxicity of (**A**) *H. italicum* infusion (HII) and (**B**) heat-killed bacteria (HKB) for proliferating Caco-2 cells. (**A**) Effect of HII on Caco-2 cell viability after 24 h of exposure. (**B**) Cytotoxicity of HKB prepared from *S.* Infantis in the exponential phase of growth after 24 h of exposure. Results are presented as mean ± RSD, *n* = 5; * *p* < 0.05, ** *p* < 0.01.

The effect of HII and HKB on the cytotoxicity of Caco-2 cells was performed by the Presto-Blue™ reagent. 24 h incubation with HII in concentrations 0.1 v/v% or lower caused no statistically significant difference in the Caco-2 cell viability (*p* > 0.05) (Figure 1A). Therefore, concentrations lower than 0.5 v/v% were used in subsequent experiments. In addition, the *S.* Infantis strain used in this study also showed no sensitivity to the tested concentrations of up to 4 v/v%.

HKB in the concentration of 2.0 × 10^8^ CFU/mL was significantly toxic for Caco-2 cells after 24 h exposure, whereas the first 2-fold dilution was no longer cytotoxic (Figure 1B). Thus, HKB in a concentration corresponding to 1.0 × 10^8^ CFU/mL of live *S.* Infantis was used for the treatment of Caco-2 cells.

### 2.2. HII Improved HKB-Induced Integrity Disruption in Caco-2 Monolayer

The TEER assay was performed to investigate whether treatment of Caco-2 cells with HII provided protective effects against integrity disruption induced by HKB. Caco-2 cells were subjected to different treatment protocols to simulate preventive action (24 h pre-treatment with HII before exposure to HKB), direct action of HII on HKB (24 h co-treatment of HII and HKB), and curative action of HII (post-treatment of cells with HII, after 24 h exposure to HKB). 

**Figure 2:**
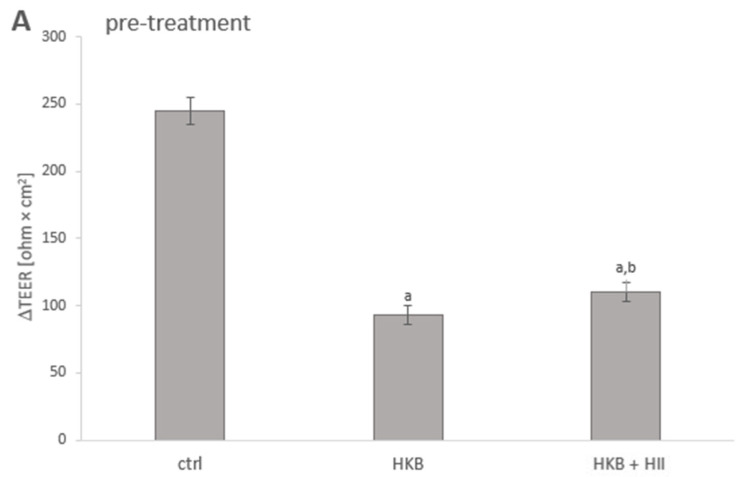

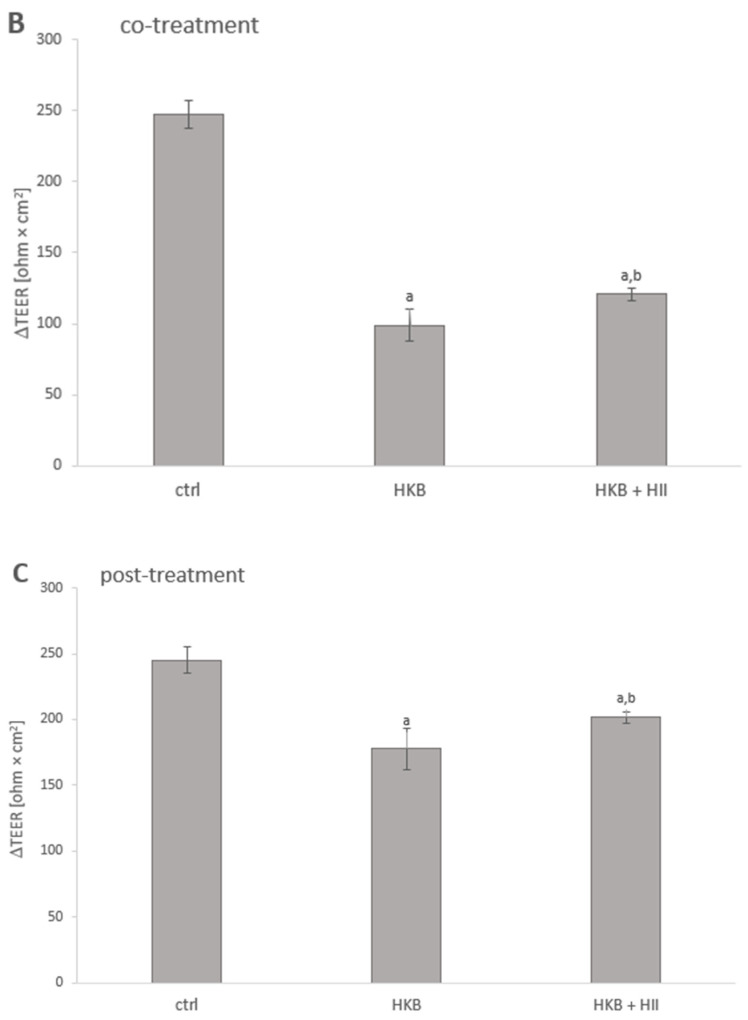
Bar graph showing changes in TEER values in (**A**) 24 h treatment with heat-killed bacteria (HKB) after 24 h pre-treatment with *H. italicum* infusion (HII), (**B**) 24 h co-treatment of Caco-2 cells with HII and HKB, (**C**) 24 h post-treatment with HII after 24 h treatment with HKB. Differences in TEER values were calculated by subtracting the values at the start of the last 24 h treatment from the values at the end of the treatment. Results are presented as mean ± SD, n = 4; a denotes a statistically significant difference (*p* < 0.05) compared to the control group, and b denotes a significant difference (*p* < 0.05) compared to the HKB-treated group.

Pre-treatment with HII for 24 h had no effect on TEER values *per se*; TEER values were 861.1 ± 19.9 ohm × cm^2^ vs. 859.6 ± 23.4 ohm × cm^2^ for control, but when followed by 24 h HKB treatment, cells pre-treated with HII had almost 10% lower decrease in TEER values compared to non-pre-treated cells (*p* = 0.0034) (Figure 2A). 24 h treatment with HKB reduced the increase in TEER of Caco-2 cells by 60.0%, while 24 h co-treatment together with HII (0.2 v/v%) resulted in a 51.3% reduction (*p* = 0.0284) (Figure 2B). Cells treated with HII (0.2 v/v%) for 24 h after being exposed to HKB for 24 h showed an increase of 9.6 % (*p* = 0.0159) in TEER values compared to media-treated cells after HKB treatment (Figure 2C). 

### 2.3. HII Improves HKB-Induced Down-Regulation of Genes Coding for Tight-Junction Proteins

Effect of different treatments with HII and HKB (pre-, co-, and post-treatment) on mRNA expressions of junctional adhesion molecule (*JAM1*), tight-junction protein-1 (*TJP1*), occludin (*OCLN*), claudin-1 (*CLDN-1*), and claudin-2 (*CLDN-2*) genes in Caco-2 cells were determined by RT-qPCR analysis.

**Figure 3:**
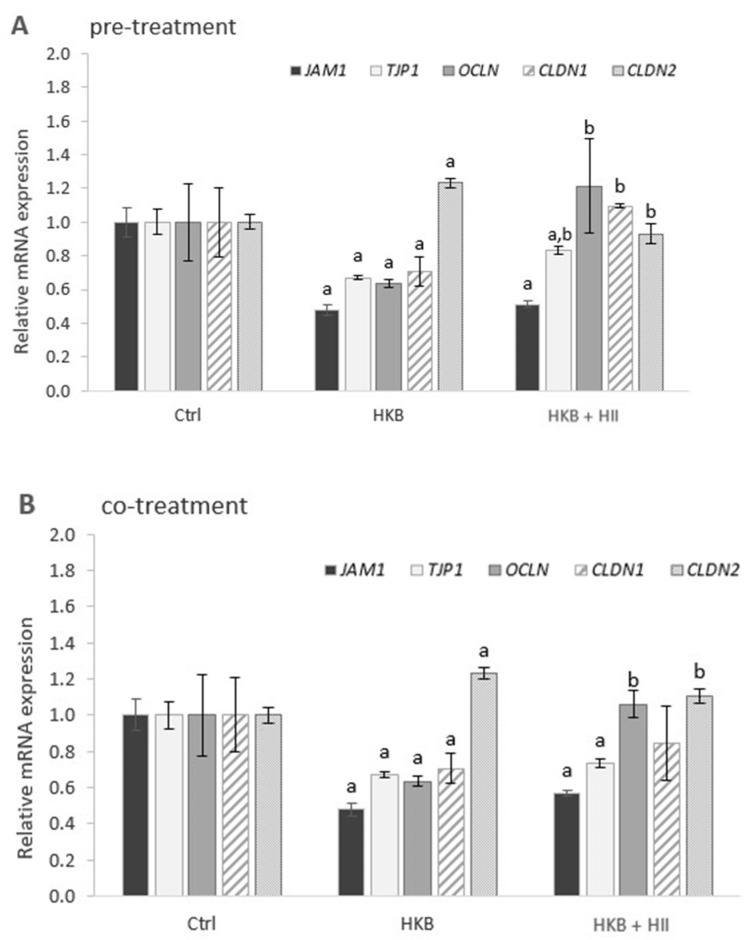

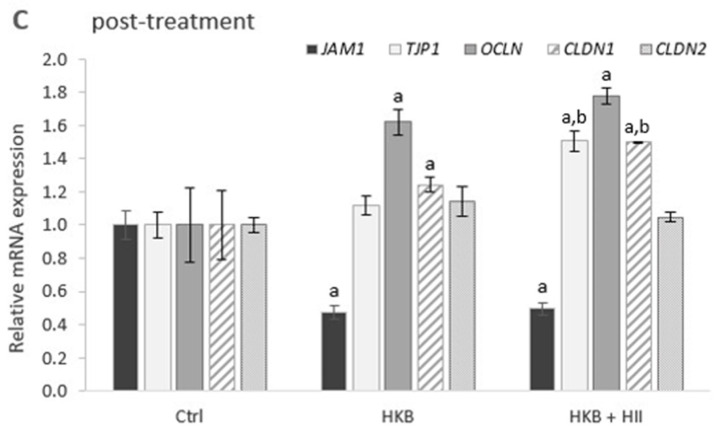
Bar graph showing relative mRNA expression of the *JAM1*, *TJP1*, *OCLN*, *CLDN-1,* and *CLDN-2* at the end of different treatments: (**A**) 24 h treatment with heat-killed bacteria (HKB) after 24 h pre-treatment with *H. italicum* infusion (HII); (**B**) 24 h co-treatment of Caco-2 cells with HII and HKB; (**C**) 24 h post-treatment with HII after 24 h treatment with HKB. Results are presented as mean ± SEM, n = 4; a denotes a statistically significant difference (*p* < 0.05) compared to the control group, and b denotes a significant difference (*p* < 0.05) compared to the HKB-treated group.

A 24 h exposure of Caco-2 cells to HKB (Figure 3A,B) resulted in a statistically significant down-regulation of all the tested genes coding for TJPs, except for *CLDN-2*, which was up-regulated. When cells were pre-treated with HII for 24 h (Figure 3A), all the tested genes except for *JAM-1* were differentially expressed compared to HKB treatment alone. The greatest effect was observed for *OCLN* and *CLDN-1,* which were even up-regulated. On the other hand, a decrease in up-regulation of *CLDN-2* was observed. A similar trend in gene expression was observed also for cells exposed to HKB and HII simultaneously (Figure 3B), however, the difference was significant only for *OCLN* and *CLDN-2*. Interestingly, 24 h after removing HKB, only *JAM-1* remained down-regulated, whereas other genes, *OCLN* and *CLDN-1* in particular, were already up-regulated (Figure 3C). Treatment with HII in this period resulted in additional up-regulation of *TJP1* and *CLDN-1*, while no statistically significant difference in expression was observed for *JAM1*, *OCLN,* and *CLDN-2* (Figure 3C). 

### 2.4. HII Reduces HKB-Induced Inflammation Response

To explore the anti-inflammatory effect of HII, alterations in gene expressions of *TNF-α*, *IL-6,* and *IL-1β* were examined by RT-qPCR after all treatments. 

**Figure 4:**
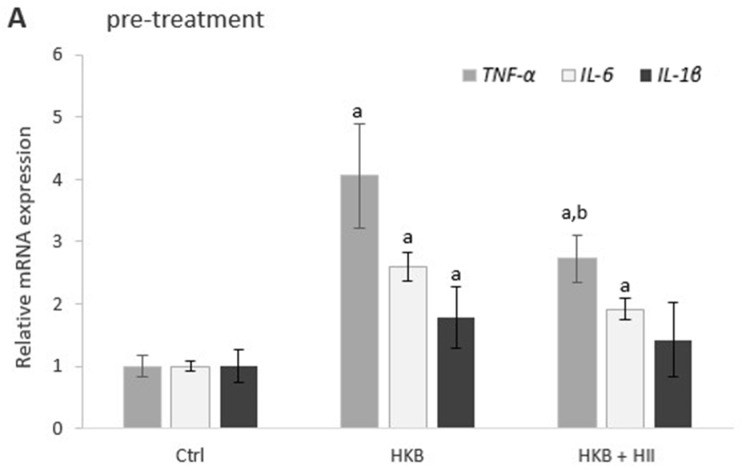

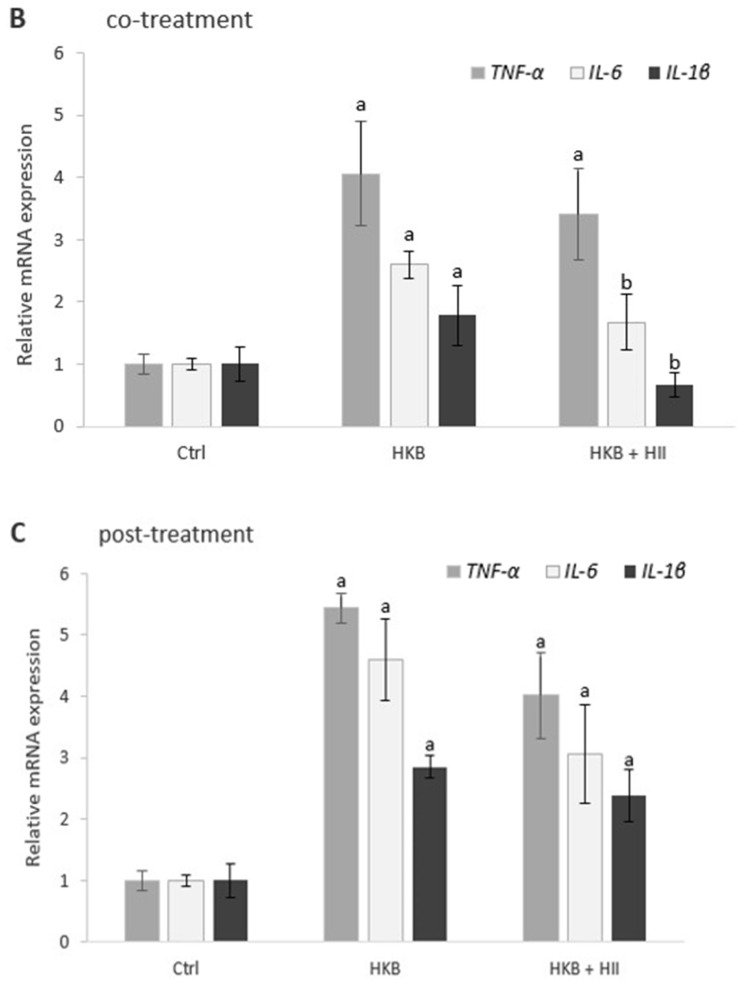
Bar graph showing relative mRNA expression of the *TNFα*, *IL-6,* and *IL-1β* at the end of different treatments: (**A**) 24 h treatment with heat-killed bacteria (HKB) after 24 h pre-treatment with *H. italicum* infusion (HII), (**B**) 24 h co-treatment of Caco-2 cells with HII and HKB (**C**) 24 h post-treatment with HII after 24 h treatment with HKB. Results are presented as mean ± SEM, *n* = 4); a denotes a statistically significant difference (*p* < 0.05) compared to the control group, and b denotes a significant difference (*p* < 0.05) compared to the HKB-treated group.

A 24 h exposure of Caco-2 cells to HKB resulted in up-regulation of all the tested inflammatory genes (Figure 4A,B), which were further up-regulated 24 h after removal of HKB (Figure 4C). Treatment with HII in this period resulted in a statistically non-significant decrease in up-regulation of *TNF-α*, *IL-6,* and *IL-1β* (Figure 4C). Co-treatment with HKB and HII caused a significant decrease in up-regulation of *IL-1β* and *IL-6* (Figure 4B), while in pre-treated cells (Figure 4A), the most significant decrease in up-regulation was observed for *TNF-α*. 

### 2.5. HII Prevents Translocation of S. Infantis

The ability of HII to mitigate the effects of *S.* Infantis on epithelial intercellular integrity was investigated on the Caco-2 cell model. In addition to the positive control (infection with *S.* Infantis alone), we conducted three comparative experiments: (a) simultaneous exposure of non-pre-treated cells to *S.* Infantis and HII; (b) *S.* Infantis infection of pre-treated Caco-2 cells; and (c) simultaneous exposure of pre-treated cells to *S.* Infantis and HII. In all experiments, TEER was measured before and 2 h post-infection. 

The integrity of cell monolayer 2 h post-infection was severely compromised with a 70.0% drop in TEER values on average, however, TEER values of cells treated with HII, regardless of the protocol used, were not significantly different when compared to non-treated cells (TEER values were 60.9 ± 12.3, 88.7 ± 7.4, 72.8 ± 30.5 and 80.3 ± 35.6 ohm × cm^2^ for positive control, (a), (b) and (c), respectively). Furthermore, the number of adhered or invaded *S.* Infantis bacteria in Caco-2 cells treated with HII was not significantly different compared to the positive control (Table 1). On the contrary, it is evident that 24 h pre-treatment with HII (0.2 v/v%) reduced the translocation of *S.* Infantis bacteria across the cell monolayer by 85%, while incubation with HII during the infection in addition to the 24 h pre-treatment completely prevented the bacterial translocation. However, treatment with HII only for the time of infection had no significant effect on bacterial translocation. 

## 3. Discussion

Intestinal epithelium provides an important barrier against bacterial translocation. Infectious intestinal pathogens, including various bacteria and viruses, have different mechanisms for gaining access to the host. Some directly adhere to and invade the intestinal epithelial barrier, whereas others disrupt this barrier via the secretion of toxins. In either case, various common pathogens have developed mechanisms that target the host’s TJPs. By disrupting the TJ complex, epithelial permeability increases, and the pathogens’ invasion process is facilitated [2]. These effectors can act directly upon constitutive TJPs through either their lytic activity leading to the degradation of TJs or through specific binding allowing the disengagement of TJPs from the junctional complex. Pathogenic factors can also trigger cell-signaling pathways involved in both TJs and cytoskeleton modulation by either inducing up- or down-regulation of gene expression or post-transcriptional events such as phosphorylation. Finally, pro-inflammatory and/or oxidative stress of intestinal epithelial cells resulting from infection can potentiate dysregulation of the TJ complex [6]. 

In the present study, we have used HKB for the disruption of the Caco-2 cell monolayer. While some studies suggest that LPS alone could induce an inflammatory response and TJP damage, other bacterial components and metabolites might also contribute. For this reason, LPS is sometimes combined with a mixture of inflammatory cytokines to achieve disruption [25,26]. In our study, treatment with HKB for 24 h caused a significant reduction of up to 60.0% in TEER increase compared to non-treated cells (Figure 2), which is therefore a suitable setting to test the potential protecting (or harming) effects of HII. A 2-h exposure to live *S.* Infantis bacteria reduced TEER much more—by up to 70.0% compared to the values before infection. Since live bacteria cannot be completely removed from cell culture and it would therefore be impossible to follow the regeneration of a monolayer, live bacteria were used only to test the effects of HII on their adhesion, invasion, and translocation. 

Caco-2 cells were subjected to three different treatment schedules: pre-, co-, and post-treatment with HII, to explore if and how HII ameliorates IB dysfunction. TEER measurements of the Caco-2 monolayer showed that the HII could improve the HKB-disrupted IB integrity. HII alone did not affect TEER, but it improved TEER when cells were exposed to HKB either before, simultaneously, or afterward. This points to both the protective role of HII as well as to its ability to stimulate the regeneration of the IB upon disruption. 

There are several possible mechanisms through which a dietary component can improve IB function: (1) by either direct interaction with TJPs or by modulating their localization, (2) by modulating the cytoskeletal structure, or (3) by increasing the expression of TJPs. Additionally, IB integrity can be enhanced indirectly through a reduction in the inflammatory response. *In vivo*, modulation of the gut microbiota represents another important mechanism for improving IB function. Based on the results of the present study, one mode of HII action is the regulation of mRNA expression of TJPs. HII improved the HKB-induced down-regulation of *TJP-1*, *OCLN*, and *CLDN-1* and decreased the up-regulation of *CLDN-2*. These effects were more evident in pre-treated cells, indicating the importance of preparing the cells for an upcoming infection/inflammatory event. Conversely, in the post-treatment protocol, 24 h after removal of HKB, *TJP-1*, *OCLN*, and *CLDN-1* were no longer down-regulated, whereas expression of *JAM-1* and *CLDN-2* was similar to the co- and pre-treatment protocols. In this case, more time has passed since the start of inflammation induction, allowing cells to initiate self-repair processes, which can also be reflected in the up-regulation of some genes coding for TJPs. Post-treatment with HII resulted in an additional increase in the up-regulation of *TJP-1* and *CLDN-1*. The changes in the expression of TJP-coding genes were in line with the expectations; occludin, TJP-1, claudin-1, and JAM-1 provide structural integrity, and therefore their up-regulation correlates with higher TEER values. Occludin has a dual role in the IB; it provides structural integrity and is an integral component in the barrier function of TJs [27]. Severely compromised occludin expression has been observed in models of intestinal inflammatory diseases, suggesting its critical role in the maintenance of IB integrity [28]. TJPs, which are peripheral membrane-associated proteins, are essential for forming scaffolds and connecting other TJPs to the cytoskeleton [3]. It has been repeatedly shown that they redistribute in nearly all types of inflammatory conditions. For example, in mammary colitis models, TJP-1 expression level in colon epithelia quickly decreased [29]. JAM-A (also known as JAM-1) is also implicated in the regulation of IB function and leukocyte migration. Mice deficient in JAM-A exhibited increased IB permeability with elevated bacterial translocation [30]. Additionally, alterations in the claudin levels can affect the IB integrity in different ways depending on the type of claudin isoform. For example, the down-regulation of claudin-1 expression level due to *CLDN-1* gene polymorphism can mediate common dysregulation of the epithelial barrier function and contribute to the progression of allergic inflammation [31]. In contrast, claudin-2, a TJP required for the formation of paracellular water channels that are highly expressed in leaky epithelial tissues, is up-regulated in inflammatory bowel diseases and promotes inflammation [32]. In our study, *CLDN-2* was in fact down-regulated upon pre- and co-treatment with HII, which indicates reduced monolayer disruption. 

The expression of some TJP genes was modulated by HII during HKB-induced barrier disruption, but since HII did not affect TEER by itself, this indicates that other mechanisms contributing to an improved IB function must be involved. It was previously shown that green tea polyphenols could enhance the intestinal epithelial immunological barrier function by inducing the secretion of antimicrobial peptides, such as β-defensins, rather than strengthening the epithelial physical barrier when no observed changes in TEER were observed [33]. Defensins are multifunctional peptides that have both antimicrobial and immunomodulatory activities. The expression of β-defensins has been associated with a proinflammatory immune response, marked by increased levels of proinflammatory cytokines through an up-regulation of *TNF-α*, *IL-6*, and *IL-8* gene transcripts [34]. The transcriptome analysis of colon fibroblasts treated with HII for 24 h indeed revealed significant down-regulation of *DEFB115* gene, which was among the most differentially expressed genes in that study [24]. The decreased expression of *DEFB115* caused by HII could thus contribute to the amelioration of inflammation response, leading to an improvement in the IB function. However, the exact role of defensin-β 115 has not been investigated yet. In the present study, HII decreased the up-regulation of *TNF-α*, *IL-1β*, and *IL-6* in all three treatment protocols, however, the most significant decrease was observed for *IL-6* and *IL-1β* in co-treated cells and for *TNF-α* in pre-treated cells. Although the inflammatory genes were the most up-regulated in cells 24 h after removal of HKB, the observed decrease in up-regulation after 24 h post-treatment with HII was not statistically significant. In agreement with this, we have shown before that participants who consumed 200 mL of HII for 28 consecutive days had reduced serum levels of IL-1β and IL-6 [23]. 

In our previous study by Kramberger et al. [24], it was observed that HII could also modulate the rearrangement of cytoskeleton and signaling through Rho GTPases. Since *Salmonella* translocates several effector proteins into host cells, among which some are known to regulate actin dynamics by modulating Rho family GTPases in order to disrupt the permeability and structure of TJs [6,35], it is possible that HII could have counteracted this action. Furthermore, it was previously shown that TJ dysfunction occurs within the first 15 min of *Salmonella* infection, suggesting its relation to early cell-signaling events and to bacterial invasion itself rather than to later downstream events such as cytokine induction [36]. In fact, in the present study, co-incubation with HII during infection in combination with 24 h pre-treatment was able to completely prevent translocation of *S.* Infantis, while pre-treatment with HII alone could hinder the bacterial translocation by 85%, but without any significant improvement in TEER. According to the current state of research, there are no known data on the effect of herbal infusions on TEER during *S.* Infantis infection, with infection studies primarily conducted on serovar Typhimurium. Virulence genes of *S.* Infantis strains, coding for different effector proteins required for host cell invasion and proteins that facilitate the internalization of bacteria into the host cell, were identified by Karacan Sever and Akan [37]. The presence of virulence genes such as *sipA*, *sipD*, *sopD*, *ssaR*, *sopB*, *sopE*, *sifA*, *and sitC* in *S.* Infantis strains was found to be similar, but notable differences were found compared to *Salmonella* Typhimurium and *Salmonella* Enteritidis strains in the presence of *sopE2*, *spvC*, and *pefA* virulence genes [37]. *In vitro* models of *S.* Typhimurium infection have revealed alterations in epithelial permeability, indicated by a reduction in TEER across infected Caco-2 cell monolayers, which facilitated bacterial translocation via the paracellular route. Furthermore, the *spvB* effector-mediated IB dysfunction was attributed mainly to the cellular redistribution of claudin-1, occludin, and E-cadherin junctional proteins [38]. In our study, bacterial translocation was prevented despite the drop in TEER values. One possible explanation is that perijunctional actomyosin contraction induced by *Salmonella* might limit the effect of dysfunctional TJs on epithelial permeability by reducing the proportion of the surface area occupied by leaky junctions [36]. It is important to note that our results show no effect of HII on the adhesion and invasion of *S.* Infantis. This is in line with other studies investigating *Salmonella* infections of Caco-2 monolayer, where the translocation defect observed by the Δ*spvB* strain had little connection to bacterial adhesion or invasion [38]. This observation further implies the HII modulation of cell-signaling processes that are important for bacterial translocation but not for bacterial adhesion and invasion.

Lastly, the improved integrity of the monolayer may also be due to the amelioration of oxidative stress. Such stress could result from HKB exposure, as inflammatory processes induce oxidative stress, characterized by an increase in the production of reactive oxygen species [39]. We have previously shown that treatment with HII up-regulates the expression of superoxide dismutase 1 in Caco-2 cells [40] and that HII is able to scavenge 83.2% of DPPH radicals [17]. There are several bioactive components present in HII, which could affect the IB function through different mechanisms and help to explain our observations. It is well known that phenolic compounds such as caffeoylquinic acids, as well as flavonoids, are potent antioxidants and anti-inflammatory agents. These compounds could have contributed to the indirect improvement of the IB dysfunction by reducing oxidative stress and inflammation, respectively. Among flavonoids, quercetin was recently found to directly modulate TJs in the respiratory mucosal barrier [41] and to improve barrier properties of the porcine small intestine [42], while kaempferol increased TEER value in IEC-6 cells and up-regulated TJP coding genes [43]. For other compounds previously determined in HII [17], such as chlorogenic acid, 5-caffeoylquinic acid, protocatechuic acid, and caffeic acid, alleviated IB disruption was observed, while chlorogenic acid could also increase TEER values [44]. It must be noted here that concentrations of individual compounds in preparations such as herbal infusions are much lower, but different compounds could contribute to additive or synergistic effects, which could thus differ from effects observed when testing the isolated substances individually. Furthermore, there are also other factors that can affect the chemical composition of an extract, such as genetic/phenotypic characteristics of the plant and its growing conditions. Antimicrobial activity was shown for several separate compounds of *H. italicum* as well as for the total extract. However, this activity can only contribute to the protective effects in the concurrent presence of bacteria and extract, therefore only in the co-treatment. Although we demonstrated that HII in tested concentrations does not inhibit *S.* Infantis, interestingly, when cells were pre-treated with HII and the HII was added concomitantly with *S.* Infantis, the translocation of bacteria was completely prevented, while just pre-treatment with HII did hinder but not prevent the translocation. On the other hand, incubation with HII only during the infection did not affect translocation compared to the positive control. This suggests that the antimicrobial activity of HII is less important than its ability to prepare the cells in advance for the upcoming infection. 

There are some limitations to this study that need to be addressed. TEER only measures the resistance of the cell monolayer and does not provide information about the structure and composition of the IB. Molecular studies, such as protein localization using immunofluorescence microscopy, would give us a better understanding of the underlying cause for the observed changes in the physiological property of the cell monolayer and help us explain how HII can prevent bacterial translocation. We have studied the expressions of both inflammatory and TJ-related genes only on the mRNA level, which does not necessarily translate into correlating proteins. In addition, other time points should be investigated to better explain the effect of HII on the modulation of inflammation since the expression of pro-inflammatory molecules is initiated already within 3–6 h after the LPS challenge [45]. 

## 4. Materials and Methods

### 4.1. Plant Material and Infusion Preparation

The plant material used in this study was *Helichrysum italicum* ssp. *italicum* (also called immortelle or everlasting) harvested from an ex situ experimental collection of the University of Primorska located near Ankaran (45°34′19.3″ N 13°46′33.2″ E) in June 2019. The material was morphologically characterized based on the presence of axillary leaf fascicles, caulinar leaf length, leaf margin, and number of capitula per synflorescence [17]. Herbarium specimen is deposited at the University of Primorska, Faculty of Mathematics, Natural Sciences, and Information Technologies, Slovenia, under the accession number HIa1_UP21.

*H. italicum* infusion (HII) was prepared by infusing 250 mg of the dried and milled plant material in 50 mL of boiling water. After a 10 min incubation, infusion was filtered through Whatman paper No. 41 and passed through a sterile 0.2 μm cellulose acetate syringe filter (Macherey-Nagel GmbH & Co. KG, Düren, Germany). Infusion prepared from the same plant material and following the same protocol was previously characterized using high-performance liquid chromatography-mass spectrometry analysis (HPLC-DAD-ESI-QTOF-MS, Agilent Technologies, Santa Clara, CA, USA) [17]. The main identified polyphenolic compounds were hydroxycinnamic acids, such as caffeic and caffeoylquinic acids and their derivatives; pyrones, including micropyrone and italipyrone; arzanol and its derivatives; and hydroxybenzoic acids. Of the flavonoids, the most abundant were quercetin and myricetin derivatives, and there were also several isobenzofuranones and acetophenones [17].

### 4.2. Preparation of Heat-Killed Bacteria

As a model of inflammation stimulation with bacterial endotoxins, heat-killed bacteria (HKB) were prepared with modifications as described before [46]. The bacteria used was *Salmonella enterica* serovar Infantis, described more in detail in Section 4.7.1. The bacterial culture in the exponential phase was diluted in distilled water and heated for 2 h at 80 °C to kill the bacteria. To confirm the inactivation of the bacteria, the heated bacterial suspension was re-cultured on Tryptic Soy Agar (TSA; Merck KGaA, Darmstadt, Germany). The suspensions were then centrifuged at 10,000 rpm for 10 min, and pellets were washed in phosphate buffered saline (PBS, Sigma-Aldrich, St. Louis, MO, USA) before being stored at −20 °C until used in subsequent experiments. Before the experiment, the effect of HKB on cell viability was determined as described in Section 4.4.

### 4.3. Cell Culture

Caco-2 colorectal cancer cells (ATCC^®^ HTB-37™, Manassas, VA, USA) were between passages 13 and 24 for all experiments and were cultured in Advanced Dulbecco’s Modified Eagle Medium (ADMEM, Gibco, Grand Island, NY, USA) supplemented with 10% Fetal Bovine Serum (FBS, Sigma-Aldrich, St. Louis, MO, USA) and 2 mM glutamine (Gibco, Grand Island, NY, USA). Cultures were maintained at 37 °C in a humidified atmosphere containing 5% CO_2_. Near confluence, cells were detached with trypsin (Sigma-Aldrich, St. Louis, MO, USA), counted, and used in experiments. 

### 4.4. Cell Viability Assay

Cell viability after exposure to HII was determined using Presto-Blue™ reagent (Invitrogen™, Carlsbad, CA, USA) according to the manufacturer’s instructions. Caco-2 cells were seeded in 96-well plates at a density of 10,000/well and cultured for 24 h. Then, cells were treated with HII or with HKB diluted in cell culture media for 24 h. PrestoBlue™ was added to each well and after 30 min, fluorescence was measured on a microplate reader, the Infinite F200 (Tecan Group Ltd., Zürich, Switzerland) at excitation/emission (ex/em) of 535/595 nm. Each experiment was carried out in five replicates.

### 4.5. Intestinal Epithelial Barrier Function Measurements

For the intestinal barrier function measurements, cells were seeded at a density of 2 × 10^5^ cells per mL onto 12 mm Transwell^®^ polycarbonate membrane inserts with 0.4 μm pore size (Corning, Inc. Lowell, MA). Transepithelial electrical resistance (TEER) was used as a measure of cell monolayer integrity. Growth media in apical and basolateral compartments was refreshed every 2–3 days. Cells were cultured until TEER values had achieved > 500 ohm × cm^2^, demonstrating a tight monolayer. After that, cells were subjected to three different treatment schedules: (1) the cells were pre-treated with HII (0.2 v/v%) for 24 h, replaced with fresh media containing HKB, and cultured for another 24 h; (2) the cells were treated with HII (0.2 v/v%) together with HKB for 24 h; and (3) the cells were first treated with HKB for 24 h, media removed, and cells washed with 500 µL of PBS, then cells were post-treated with fresh media containing HII (0.2 v/v%) for another 24 h.

TEER was assessed based on the manufacturer’s instructions and previously described approaches [47] before and after all treatments. TEER was measured using an epithelial volt–ohm meter with a chopstick electrode (Millicell ERS-2, EMD Millipore, Billerica, MA, USA). The electrode was immersed at a 90° angle with one tip in the basolateral chamber and the other in the apical chamber. Care was taken to avoid electrode contact with the monolayer, and triplicate measurements were recorded for each monolayer. An insert without cells was used as a blank and its mean resistance was subtracted from all samples. Unit area resistance was then calculated by multiplying resistance values by the effective membrane area (1.12 cm^2^).

### 4.6. RNA Isolation and RT-qPCR Analysis

At the end of IB integrity measurements, RNA was isolated from treated and control Caco-2 cells using QIAzol^®^ Lysis Reagent (Qiagen N.V., Hilden, Germany) following the manufacturer’s instructions. Isolated RNA was quantified with the Multiskan Sky spectrophotometer (ThermoFisher Scientific, Waltham, MA, USA), and its quality was evaluated based on A_260_/_280_ and A_260_/_230_ ratios. Two µg of RNA were then reverse transcribed to cDNA with a QuantiTect^®^ Reverse Transcription kit (Qiagen N.V., Hilden, Germany).

Real-time quantitative polymerase chain reaction (RT-qPCR) was performed using QuantiNova™ SYBR^®^ Green PCR Kit (Qiagen N.V., Hilden, Germany) and 50 ng of cDNA template on a QuantStudio^®^ 3 Real-Time PCR System (Thermo Fisher Scientific, Waltham, MA, USA) according to the manufacturer’s protocol. Primers for tumor necrosis factor-α (*TNF-α)*, interleukin-1β (*IL-1β*), interleukin-6 (*IL-6*), tight junction protein-1 (*TJP1*), claudin-1 (*CLDN1*), claudin-2 (*CLDN2*), occludin (*OCLN*), junctional adhesion molecule A (*JAM1*), and glyceraldehyde-3-phosphate dehydrogenase (*GAPDH)* were selected from PrimerBank [48] and manufactured at 25 nmol scale by Integrated DNA Technologies (Coralville, IA, USA). In RT-qPCR reactions, primers were used at a 0.7 µM final concentration. Details of the PCR primers are listed in Table 2. 

PCR reaction conditions were 95 °C for 2 min and 40 cycles of 95 °C for 5 s and 60 °C for 10 s. Melting curves were inspected to ensure primer specificity, and the amplification curves of the qPCR products were analyzed with the QuantStudio™ Design and Analysis software v1.5.0 using the comparative cycle threshold (C_t_) method. C_t_ values of all the targeted genes were normalized to *GAPDH*, which was selected as a reference gene after verification of maintained expression across treatments, as previously validated in Caco-2 cells [49]. The results are presented as the fold change (−ΔΔC_t_) compared to non-treated cells.

### 4.7. Adhesion, Invasion, and Translocation of S. Infantis 

#### 4.7.1. Bacterial Culture 

For the infection assay, *Salmonella enterica* serovar Infantis (*S.* Infantis) 323/19 strain originating from poultry from the *Salmonella* strain collection of the Slovenian Veterinary Faculty, Institute of Microbiology and Parasitology was used. The strain was previously characterized by whole-genome sequencing [50] and exhibits resistor type as follows: *tet*(A), *aac*(6′)-*Iaa*, *aadA1*, *sul1*, *parC* (T57S), *gyrA* (S83Y), *nfsA* (NS159). It also harbors the plasmid of emerging *S.* Infantis (pESI).

The working bacterial culture was prepared by inoculating 5 mL of Tryptone Soya Broth (TSB; Oxoid Ltd., Hampshire, UK) with a single Colony Forming Unit (CFU) and then incubated at 37 °C with shaking at 160 rpm for 3 h. For the experiments, the bacterial suspension in exponential growth was diluted in the ratio of 1:5 in sterile distilled water to obtain HKB or in ADMEM for the infection assay. Prior to the infection assay, the antimicrobial effect of HII against *S.* Infantis was determined as described before [51].

#### 4.7.2. Infection Assay

Adhesion, invasion, and translocation of *S.* Infantis to the basolateral compartment of the well were determined in a functional cell model using Caco-2 cells as previously described [52]. Briefly, after the 10 day incubation time, the medium covering the Caco-2 cells was replaced by fresh ADMEM or the medium containing 0.2 v/v% of HII. After the 24 h incubation with HII or medium, the cell monolayers were washed once with 100 μL PBS, and infection assays were performed by seeding the bacterial inoculum of *S.* Infantis culture in the exponential growth (approximately 2 × 10^8^ CFU/mL) after washing step and replacement of TSB with ADMEM. The infected cells were then incubated at 37 °C in 5% CO_2_ for 2 h, to allow adhesion and invasion. The total numbers of adhered *S.* Infantis were determined at 2 h post-infection after lysing the cells by the addition of 500 μL 0.1% Triton^TM^ X-100 (Sigma-Aldrich, St. Louis, MO, USA) for 10 min. The number of bacteria was determined by plating the serially diluted suspension on TSA (Merck, KGaA, Darmstadt, Germany). To determine the number of invaded *S.* Infantis bacteria, the cells were washed with PBS and incubated for 1 h with 400 μg/mL gentamicin (Sigma-Aldrich, St. Louis, MO, USA). The monolayers were then washed twice with PBS and lysed by adding Triton^TM^ X-100. The number of adhered cells was calculated by subtracting the number of cells before and after gentamicin treatment. In addition, the numbers of translocated *S.* Infantis were determined simultaneously. To investigate the effect of HII on Caco-2 cell monolayer integrity following the infection, the TEER was also measured before and 2 h post-infection. 

### 4.8. Statistical Analysis

The results were expressed as mean values ± standard deviation or as mean values ± standard error of the mean. An independent sample *t*-test for normally distributed variables and Mann–Whitney U for non-normally distributed variables were used to compare the differences between differently treated cells. All statistical outcomes with *p* values less than 0.05 (*p* < 0.05) were recognized as statistically significant. Statistical analyses were performed with the help of computer software—Statistical Package for the Social Sciences (SPSS) version 23.0 (IBM Inc., Chicago, IL, USA).

## 5. Conclusions

IB dysfunction contributes to the pathology of inflammatory bowel disease and metabolic disorders, which are growing public health problems. Dietary components and nutritional supplements, including herbal beverages, have demonstrated the ability to provide clinically important reductions in circulating endotoxins and improve related sequelae, such as inflammation and other negative health markers. Our study demonstrated that HII could indeed improve the inflammation-induced IB dysfunction through indirect up-regulation of TJP1, occludin, and claudin-1 and down-regulation of claudin-2 mRNA expression in the Caco-2 cell IB model. The most profound effect on the IB integrity was observed in pre-treated Caco-2 cells; therefore, it can be recommended to consume HII in a preventive manner. In addition, 24 h pre-treatment with HII was also able to prevent the translocation of live *S.* Infantis. HII could probably be effective also curatively, but longer consumption of HII would be needed to repair the damage caused by the inflammatory agents. Overall, these results have broadened our understanding of the HII modulation of the IB integrity and confirmed once again, the health-beneficial effects of HII consumption in mild inflammatory conditions. However, further studies are needed to confirm other possible mechanisms discussed in this article.

## Figures and Tables

**Table 1:** Number (log CFU/mL) of adhered, invaded *S.* Infantis 323/19 bacteria, and the number of translocated bacteria to the basolateral compartment.

	Positive Control (S. Inf)	(a) S. Inf and HII	(b) 24 h HII + S. Inf	(c) 24 h HII + S. Inf and HII
Adhesion	1.67 ± 0.30	1.74 ± 0.19	1.65 ± 0.30	1.63 ± 0.30
Invasion	5.99 ± 0.18	6.07 ± 0.27	6.31 ± 0.30	6.02 ± 0.17
Translocation	3.10 ± 0.52	3.49 ± 0.19	0.44 ± 0.88 *	0 *

Data are presented as mean ± SD of log CFU/mL; * denotes a statistically significant difference (*p* < 0.05) compared to the positive control. Legend: (a) *S.* Infantis infection together with HII without prior cell pre-treatment; (b) *S.* Infantis infection of Caco-2 cells pre-treated with HII for 24 h; (c) *S.* Infantis infection together with HII of 24 h pre-treated cells.

**Table 2:** Sequences of primers used for quantitative real-time PCR.

Gene Name	Primer Sequence
*TNF-α*	F: 5′-CCTCTCTCTAATCAGCCCTCTG-3′
	R: 5′-GAGGACCTGGGAGTAGATGAG-3′
*IL-6*	F: 5′- ACTCACCTCTTCAGAACGAATTG-3′
	R: 5′-CCATCTTTGGAAGGTTCAGGTTG-3′
*IL-1β*	F: 5′-ATGATGGCTTATTACAGTGGCAA-3′
	R: 5′-GTCGGAGATTCGTAGCTGGA-3′
*TJP1*	F: 5′-CAACATACAGTGACGCTTCACA-3′ R: 5′-CACTATTGACGTTTCCCCACTC-3′
*CLDN1*	F: 5′-GGCAACTAAAATAGCCAGACC-3′ R: 5′-CCTCCTGGGAGTGATAGCAAT-3′
*CLDN2*	F: 5′-CGGGACTTCTACTCACCACTG-3′ R: 5′-GGATGATTCCAGCTATCAGGGA-3′
*OCLN*	F: 5′-ACAAGCGGTTTTATCCAGAGTC-3′ R: 5′-GTCATCCACAGGCGAAGTTAAT-3′
*JAM1*	F: 5′-ACCTTCTTGCCAACTGGTATCA-3′ R: 5′-AGCACGATGAGCTTGACCTTG-3′
*GAPDH*	F: 5′-GGAGCGAGATCCCTCCAAAAT-3′ R: 5′-GGCTGTTGTCATACTTCTCATGG-3′

## Data Availability

The original contributions presented in the study are included in the article, further inquiries can be directed to the corresponding author.
